# Geometric Angles and Gene Expression in Cells for Structural Bone Regeneration

**DOI:** 10.1002/advs.202304111

**Published:** 2023-09-29

**Authors:** Juan Wang, Qianhao Yang, Qimanguli Saiding, Liang Chen, Mingyue Liu, Zhen Wang, Lei Xiang, Lianfu Deng, Yixuan Chen, Wenguo Cui

**Affiliations:** ^1^ Department of Orthopaedics Shanghai Key Laboratory for Prevention and Treatment of Bone and Joint Diseases Shanghai Institute of Traumatology and Orthopaedics Ruijin Hospital Shanghai Jiao Tong University School of Medicine 197 Ruijin 2nd Road Shanghai 200025 P. R. China; ^2^ Department of Orthopedic Surgery Shanghai Jiao Tong University Affiliated Sixth People's Hospital Shanghai 200233 P. R. China

**Keywords:** bone regeneration, geometric angles, microfibers, microRNA sequencing, stem cell functions

## Abstract

Geometry and angles play crucial roles in cellular processes; however, its mechanisms of regulation remain unclear. In this study, a series of three dimensional (3D)‐printed microfibers with different geometries is constructed using a near‐field electrostatic printing technique to investigate the regulatory mechanisms of geometry on stem cell function and bone regeneration. The scaffolds precisely mimicked cell dimensions with high porosity and interoperability. Compared with other spatial topography angles, microfibers with a 90° topology can significantly promote the expression of osteogenic gene proteins in bone marrow‐derived mesenchymal stem cells (BMSCs). The effects of different spatial structures on the expression profiles of BMSCs differentiation genes are correlated and validated using microRNA sequencing. Enrichment analysis shows that the 90° microfibers promoted osteogenesis in BMSCs by significantly upregulating miR‐222‐5p/cbfb/Runx2 expression. The ability of the geometric architecture to promote bone regeneration, as assessed using the cranial defect model, demonstrates that the 90° fiber scaffolds significantly promote new bone regeneration and neovascular neural network formation. This study is the first to elucidate the relationship between angular geometry and cellular gene expression, contributing significantly to the understanding of how geometric architecture can promote stem cell differentiation, proliferation, and function for structural bone regeneration.

## Graphical abstract

A series of microfibers with different geometries are constructed to investigate the regulatory mechanisms of geometry on stem cell function and bone regeneration. The microfiber patterns with a 90° topology can efficiently promote the expression of osteogenic gene proteins of mesenchymal stem cells through the upregulation of the miR‐222‐5p/cbfb/Runx2 axis, accelerating new bone regeneration and neurovascular networks formation.

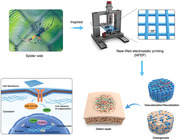

## 1 Introduction

Angularity is a typical natural topology. For example, many plants have leaves with an arc angle of 137.5°. At an angle of 137.5°, leaves optimally occupy space and are exposed to increased sunlight and rain.^[^ ^1^ ^]^ A computer simulation by Vogel found that sunflower fruits on a disk were arranged in a tightly packed and uniform manner, accommodating the highest number of fruits, only when the dispersion angle was 137.5°. Experimental studies have shown that such phenomena are caused by the long‐term influence of Earth's magnetic field on plants.^[^ ^2^ ^]^ Furthermore, many animal “mathematicians” are found in nature. The octagonal geometric pattern of spider webs is both complex and homogeneous, with well‐defined radii, chords, parallel segments, triangles, congruent corresponding angles, logarithmic spirals, suspended chain lines, and transcendental lines. Notably, there are many geometric angles and structures in natural bone tissue; the femoral neck has an anterior inclination and neck‐shaft angle of 15° and 130°, respectively. These angles are inherent and abnormalities in these angles cause limb deformities and dysfunction.^[^ ^3^ ^]^ Type H blood vessels at the epiphysis have a tortuous morphology that provides more nutrition to the epiphyseal plate^[^ ^4^ ^]^; the Haversian system, which carries blood vessels and nerves, is parallel to the axis of the long diaphysis, while Volkman's canal is perpendicular to the diaphysis, together forming a complex system of vascular networks in the bone tissue.^[^ ^5^ ^]^

Cells are experts in deformation geometry and can perceive the shape of their surroundings to make self‐adjustments. Therefore, geometry plays a vital role in cellular responses. For example, in the human brain, nerve cells are interconnected to form geometrically shaped neural networks to conduct signals.^[^ ^6^ ^]^ Recently, researchers discovered that epithelial cells can form a shield‐like structure on the skin, organs, and blood vessels as a natural barrier.^[^ ^7^ ^]^ The orderly arrangement of cells within human tissues endows them with specific functions, and the progression of abnormal geometries can have devastating effects on tissue function. The altered geometry of collagen fibers observed in osteoarthritis, with irregular alignment, can amplify local tissue strain to stress, leading to further damage and cell death in superficial areas.^[^ ^8^ ^]^ The altered alignment of stem cells in tendon‐bone grafts can lead to the misdirection of their differentiation and ultimately to the development of ossifying tendonitis.^[^ ^9^ ^]^ The occurrence of such diseases emphasizes the importance of geometric angles in tissues to maintain “correct” cellular function. Moreover, an in‐depth insight into the role of geometric angles in cells and tissues may contribute to our understanding of physiological homeostasis and cellular biological behavior. Recently, it has been shown that surface geometry (concave/convex) has a significant effect on the regulation of stem cell behavior and function, with convex surfaces potentially promoting the osteogenesis of bone marrow‐derived mesenchymal stem cells (BMSCs), deforming nuclei, and increasing laminin A levels. Furthermore, the mean curvature of curved surfaces determines the cell shape and growth behavior by regulating cell contractility.^[^ ^10^ ^]^ These studies indicate the significance of geometry in regulating cellular behavior; however, the current studies are limited to the behavior of cells in response to changes in geometry. The regulatory mechanisms triggered by different geometries that specifically regulate the recruitment, proliferation, and differentiation of different cell types, which in turn affect the tissue repair process, remain largely unexplored.

**Scheme 1:**
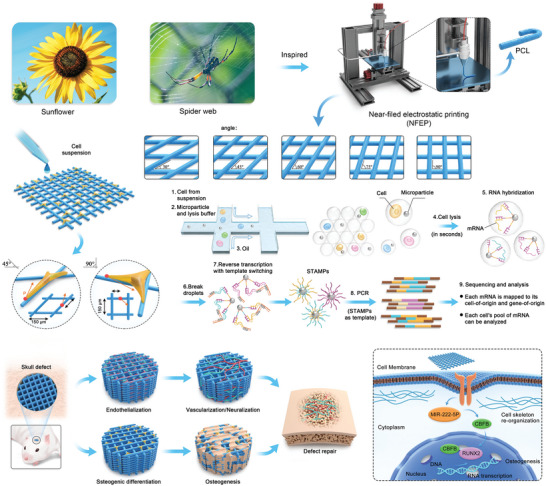
Schematic illustration of the systemic correlation of angularity and cell differentiation process.

To systematically investigate the correlation between angularity and cell behavior, we employed near‐field electrostatic printing to construct fibrous scaffolds with topological angles of 30°, 45°, 60°, 75°, and 90°. These scaffolds were co‐cultured with BMSCs, human umbilical vein endothelial cells (HUVECs), and Schwann cells. We found that the differentiation destiny was significantly correlated with the angles of the structures. Furthermore, by employing microRNA (miRNA) and mRNA sequencing, we explored the molecular mechanisms connecting different geometric angles and the regulation of cell behavior (**Scheme** **1**). Moreover, we systematically interpreted the effects of different geometric angles on bone repair by implanting 45° and 90° fiber scaffolds into bone defect models using micro‐computed tomography (micro‐CT), and morphological and histological analyses.

## 2 Results and Discussion

### 2.1 Design of Scaffolds

The development of 3D printing technology has made the design of the spatial geometry and internal structure of biological materials more convenient. 3D scaffold materials can be fabricated according to the specialized requirements of the tissue structure and can be applied to the repair of bone defects, blood vessels, nerves, and other macro‐and microtissues. Especially in bone injury, 3D scaffold materials can be used to design structures according to the injury types, bone shapes, and other factors, and can be applied to bone injury repair under different conditions.^[^ ^11^ ^]^ 3D scaffolds with different components have been applied in bone injury repair to provide mechanical support or biomimetic structures, and have shown good bone formation effects.

**Figure 1:**
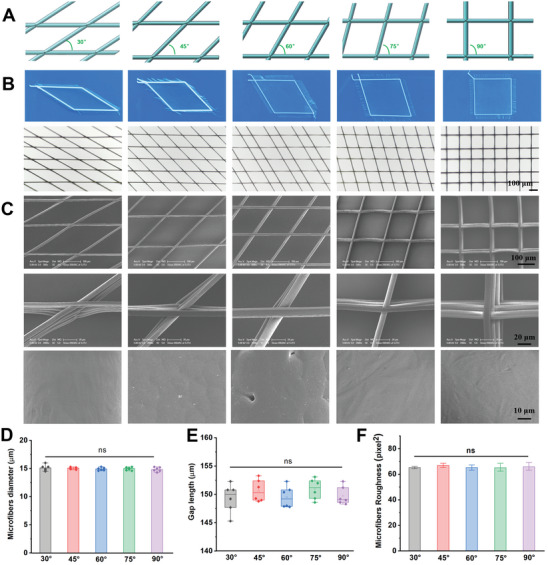
Characterization of 3D microfibers with different overlay angles from 30° to 90°. A) Schematic diagram of different microfibers; B) Microscopy characterization of various microfiber patterns at different magnifications, scale bar: 100 µm; C) Representative SEM images of the microfiber patterns at high magnification; D) The statistics of microfiber diameter; E) The statistics of print gap length; F) The statistics of surface roughness of the microfiber patterns. The data are presented as mean ± SD, *n* = 6, and ^**^ *P* < 0.01.

However, millimeter‐scale fiber sizes are not conducive to the structural remodeling of the cellular microenvironment, limiting the applicability of constructing fiber microenvironments to study cell behavior. The development of near‐field electrostatic printing (NFEP) has overcome the shortcomings of the conventional 3D printing fiber scales and the unsteady whips of the electrospinning jet and helps to prepare controlled fiber structures with different geometric angles at the cellular level (that is, at the micron level).^[^ ^12^ ^]^ The wide potential applications of the electro‐writing scaffold makes it an ideal technology for studying the controllable structure of cell behavior, which makes it conducive to accurately exploring the regulatory effects of the composition, structure, performance, and other factors of bionic fiber scaffolds on cell function. Further, it can aid our understanding of the mechanisms of action and tissue regeneration effects at the molecular, cellular, and tissue levels. However, cellular regulation of the symbiotic ecological niche formed by the activation of 3D‐printed scaffolds and structures from different geometric angles has not been systematically studied. Additionally, the effects of these structures on stem cell behavior and functional bone regeneration remain unknown. Therefore, fiber scaffolds with topological angles of 30°, 45°, 60°, 75°, and 90° were constructed from polycaprolactone (PCL) at the cellular level using NFEP technology to explore the mechanism underlying the influence of geometric angles on the biological behavior of cells (**Figure** **1**A).

To investigate the response of functional cells to different interfiber overlay angles of microfibers, NFEP was used to construct 3D structures with fiber angles between 30° and 90°, fiber diameters of 15 µm (similar to cell sizes), and the average distance between the fibers (gap length) of 150 µm (Figure 1B). The appearance and topography of microfibers with different interfiber overlay angles were observed using digital photographs and scanning electron microscopy (SEM). SEM images at vary magnifications exhibited that all groups presented microfibrous structures composed of cross‐oriented fibers at different angles, consistent with their original design (Figure 1C–E), their surfaces were homogeneous and smooth without obvious rough and granular structures (Figure 1F; Figure S1, Supporting Information), thus ensuring that the angle between fibers is the single factor regulating cell function, which lays a foundation for the subsequent cellular experiments.

### 2.2 The Biological Behavior of HUVECs

At present, vascularization of artificial grafts is one of the greatest challenges in complex tissue regeneration.^[^ ^13^ ^]^ The network of blood vessels in tissues and organs transmits oxygen and nutrients, maintaining metabolism and other biological activities of the body. Endothelial cells are essential vascular cells that respond to physical or chemical external signals and perform a series of cellular activities, including proliferation, migration, and vascular morphogenesis. Cell proliferation and behavioral assays were performed on HUVECs to evaluate the effects of different interfiber overlay angles.

**Figure 2:**
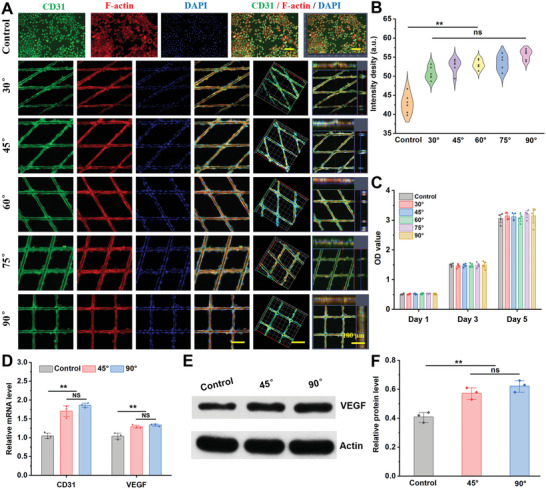
Biological behavior of HUVECs cultured on different 3D microfibers with different overlay angles from 30° to 90°. A) Representative fluorescence images of HUVECs after 5 d of culture at different magnifications, scale bar: 100 µm; B) Fluorescence intensity of CD31 acquired from (A) Immunofluorescence staining. The data are presented as mean ± SD, *n* = 6, and ^**^ *P* < 0.01; C) The proliferation of HUVECs after 1, 3, and 5 d cultured on control and different microfibers; D) RT‐PCR results of CD31 and VEGF from the HUVECs on various groups. The data are presented as mean ± SD, *n* = 3, and ^**^ *P* < 0.01; E) Western blotting bonds of VEGF and Actin from different groups and F) quantitative analysis of protein expression. The data are presented as mean ± SD, *n* = 3, and ^**^ *P* < 0.01.

After 5 d of cell culture, HUVEC morphology and vascular network behavior were evaluated by cytoskeletal and immunofluorescence staining (**Figure** **2**A). The results showed that the cells presented a well‐stretched morphology on all microfibers and could wrap the fibers in directional extension growth, whereas the cells in the control group showed irregular growth. Notably, CD31 immunofluorescence intensity confirmed that the CD31 secretion from HUVECs on microfibers was significantly higher than that from the control group. However, there was no significant difference between the material groups (Figure 2B). The CCK‐8 assay showed that cells continued to proliferate over prolonged culture durations, and there was no statistical difference between microfibers, indicating that the fibers were not cytotoxic and that the change in fiber angle had no significant effect on cell proliferation (Figure 2C).

The ability of scaffolds to promote vascular formation plays an important role in bone regeneration because neovascularization can accelerate bone repair by delivering nutrients and oxygen to the site of injury.^[^ ^14^ ^]^ The ability of various fibers to induce vascularization was studied via reverse transcription‐quantitative real‐time polymerase chain reaction (qRT‐PCR) and western blotting. As shown in Figure 2D, the VEGF and CD31 expression in the fiber groups was significantly higher than that in the control group (*P* < 0.01), however, there was no significant difference between the fiber groups. Similar results were obtained by western blotting (Figure 2E,F). These results further suggest that the microfibers group could effectively promote endothelial cell angiogenesis compared to the control group.

### 2.3 Biological Behavior of SCs and PC12

**Figure 3:**
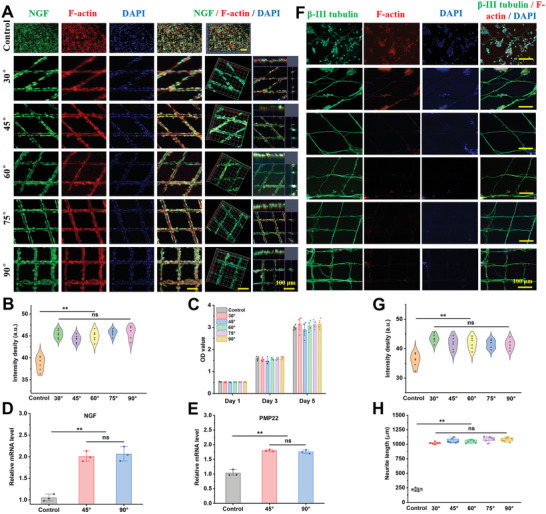
Biological behavior of SCs and PC12 cells cultured on different 3D microfibers. A) Representative immunofluorescence images of SCs at 5 d; SCs stained with NGF (green), F‐actin (red), and DAPI (blue) at different magnification, scale bar: 100 µm; B) Fluorescence intensity of NGF acquired from (A) Immunofluorescence staining. The data are presented as mean ± SD, *n* = 6, and ^**^ *P* < 0.01; C) SCs proliferation cultured on control and various microfibers for 1, 3, and 5 days; D,E) RT‐qPCR analysis of NGF and PMP22 gene expressions from the SCs on various groups. The data are presented as mean ± SD, *n* = 3, and ^**^ *P* < 0.01; F) Representative immunofluorescence images of PC12 cells after 21 d, and the cells were stained with β‐tubulin III (green), DAPI (blue), and F‐actin (red), scale bar: 100 µm; G) Fluorescence intensity of β‐tubulin III and H) the quantitative analysis of neurite length acquired from (B). The data are presented as mean ± SD, *n* = 6, and ^**^ *P* < 0.01.

Recent studies have reported that the development of micronerves in bone is a prerequisite for bone development and maturation during embryonic development.^[^ ^15^ ^]^ Histological studies have shown that blood vessels and nerves in bone often accompany each other.^[^ ^16^ ^]^ During fracture healing, neuronal axons secrete various factors that guide blood vessel growth and bone regeneration. Neuroglia (Schwann cells, SCs) were selected to investigate the response of nerve cells to different angle topologies during injury repair. SCs grew into a disordered state on the culture plate and exhibited a unique anisotropy on the cell scales with a diameter of 15 µm. The ability of the fibers to guide cell growth did not change with increasing topological angles on the fiber surface (**Figure** **3**A). According to the immunofluorescence staining analysis, cell morphology also changed significantly from the immature round to the mature bipolar state compared with the control cells. Immunofluorescence intensity analysis showed that the microfiber groups were more conducive to the expression of F‐actin and nerve growth factor (NGF) in the cells (Figure 3B). Cell proliferation experiments indicated that microfibers from different angles significantly promoted cell proliferation and growth compared to the control group, but there was no statistical difference between the fiber groups (Figure 3C). RT‐qPCR also confirmed that microfibers were more conducive to the expression of SC functional genes, such as PMP22 and NGF (Figure 3D,E), which may be attributed to the fact that microfibers are more conducive to maintaining the balance of cell‐cell interactions and cell‐matrix interactions, compared with the control group.

Given the local guidance of neurite growth by the micropatterns, we were interested in understanding how the angle of coverage between the microfibers affects neurite expansion. When PC12 cells were cultured at different microfibers, it was found that most of the neurites extended along the microfibers and were closely related to the underlying pattern at all angles (Figure 3F). Quantification of the fluorescence intensity of β‐tubulin III and neurite length suggested no significant differences between the different angle groups, but they significantly differed from the control group (Figure 3G,H).

### 2.4 Biological Behavior of BMSCs

**Figure 4:**
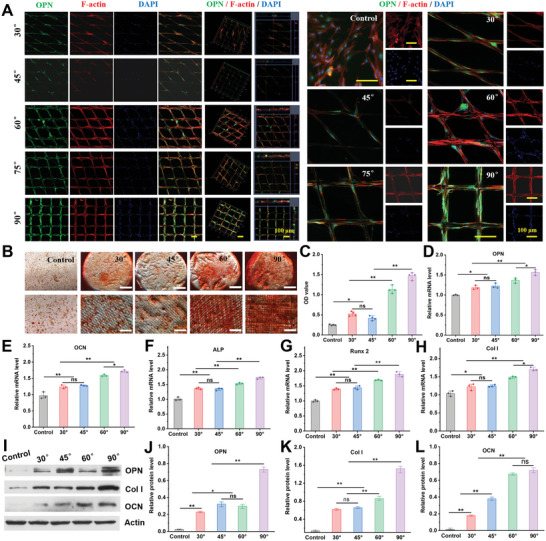
Biological behavior of BMSCs cultured on different 3D microfibers. A) Representative immunofluorescence images of BMSCs at 14 d. BMSCs stained with OPN (green), DAPI (blue), and F‐actin (red) at different magnification, scale bar: 100 µm; B) ARS staining of BMSCs cultured in different 3D microfibers, scale bar: 2 mm and 200 µm; C) ARS activity assay of the BMSCs on the different groups after 21 d of culture. The data are presented as mean ± SD, *n* = 4, ^*^ *P* < 0.05, and ^**^ *P* < 0.01. D–H) RT‐qPCR analysis of Osteogenesis‐related genes expression from the BMSCs on various groups after 14 d of culture. The data are presented as mean ± SD, *n* = 3, ^*^ *P* < 0.05, and ^**^ *P* < 0.01; I) Representative western blotting bonds for OPN, Col I, OCN, and Actin of the BMSCs on various groups after 14 d of culture and J–L) quantitative analysis of protein expression obtained from Western blotting (I). The data are presented as mean ± SD, *n* = 3, ^*^ *P* < 0.05, and ^**^ *P* < 0.01.

Stem cells play an important role in tissue regeneration and are used extensively. BMSCs are widely used for bone tissue repair during bone regeneration.^[^ ^17^ ^]^ To investigate the effect of the microfiber patterns on the osteogenic differentiation of BMSCs, OPN immunofluorescence staining, Alizarin Red S (ARS) staining, and RT‐PCR were used. As a key marker of osteogenesis, OPN immunofluorescence staining was carried out at 14 d of culture, which revealed that the most efficient enhancement of osteogenic differentiation was in microfibers‐90°, in which the relative OPN expression increased, compared with other angles and the control (**Figure** **4**A), indicating that 90° microfibers promoted the osteogenic differentiation of BMSCs. ARS staining was used to evaluate the effect of various microfibers on bone marrow mesenchymal stem cell differentiation, as calcium nodules are important markers of advanced osteogenesis. Denser and higher numbers of calcium nodules were found in the 90° microfiber group, indicating they have higher osteogenic activity (Figure 4B). Further quantification of ARS staining and calcium nodules demonstrated that 90° microfibers had higher osteogenic activity than the other microfibers (Figure 4C). A scaffold requires good cytocompatibility to be applied in vivo, so we evaluated the cytocompatibility of the microfibers. In addition, RT‐PCR was performed to assess the expression of five genes: OPN, OCN, ALP, Runx2, and Col I. As shown in Figure 4D–H, the expression of these genes was higher in the 90° microfibers group than in the other groups. Similar results were obtained using western blotting (Figure 4I–L). These results demonstrate that 90° microfibers had better osteogenic potential than the other microfibers.

### 2.5 Mechanism of Microfibers in Bone Regeneration

**Figure 5:**
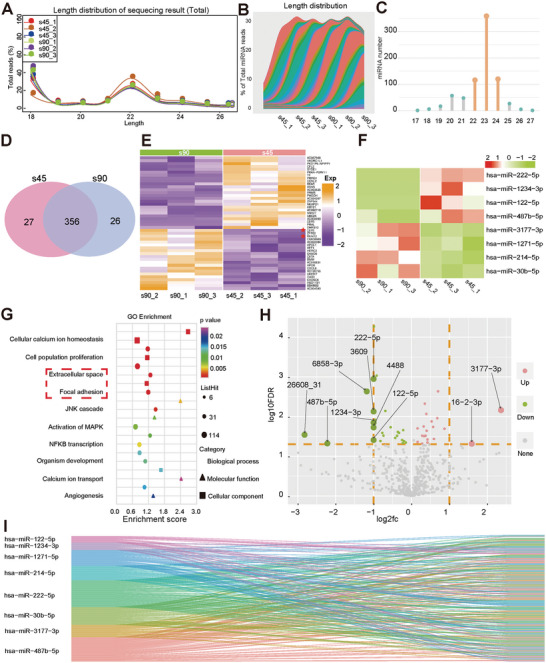
The identification of miR‐222‐5p as the mediator to strengthen the osteogenic ability. A,B) The length distribution of miRNAs of each group in sequencing result (s45 means sample of 45° microfibers group, and s90 means sample of 90°microfibers group, hBMSCs were co‐cultured with s45 or s90 in osteogenic‐inducing medium for 24 h, three replicates for each group); C) The length distribution of miRNAs of all six groups; D) The number of differential miRNAs in sequencing result in either s45 or s90 group; E) The heatmap of 50 differential mRNAs revealed by mRNA sequencing (*n* = 3, downregulation: yellow color, fold change < 0.5, ^*^ *p* < 0.05; upregulation: purple color, fold change > 1.5, ^*^ *p* < 0.05); F) The heatmap of eight differential miRNAs revealed by miRNA sequencing (*n* = 3, downregulation: yellow color, fold change < 0.5, ^*^ *p* < 0.05; upregulation: purple color, fold change > 1.5, ^*^ *p* < 0.05); G) Go enrichment analysis of miRNA sequencing result indicated extracellular space and focal adhesion are among the most related biological process; H) The scatter diagram indicated the fold change of eight differential miRNAs. I) The corresponding mRNA of eight differential miRNAs.

The expression profiles of different genes associated with each spatial structure in BMSCs were correlated and validated using western blotting and ARS, and enrichment analysis showed that the 90° microfibers promoted the osteogenesis of BMSCs most significantly. To explore the underlying molecular mechanism, miRNA sequencing was performed on the hBMSCs (45° and 90° microfiber groups, *n* = 3). The length distribution of the miRNAs was primarily between 22 and 24 bp in each group (**Figure** **5**A–C). Figure 5D presents a Venn diagram illustrating the number of differentially expressed miRNAs. Sequencing revealed eight differentially expressed miRNAs (Figure 5F; upregulation: fold change > 1.5, downregulation: fold change < 0.5, ^*^ *P* < 0.05; ^*^ *P* < 0.05). Kyoto Encyclopedia of Genes and Genomes (KEGG) and Gene Ontology (GO) pathway analyses of the miRNA sequencing results revealed that both extracellular matrix (ECM)‐receptor interactions and focal adhesions were involved (Figure 5G; Figure S2, Supporting information).

**Figure 6:**
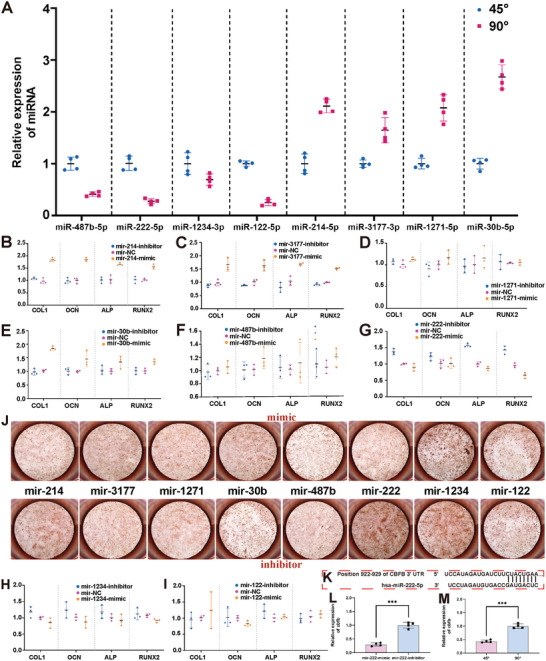
The verification of target miRNA. A) Verification of the expression of eight differential miRNAs via qPCR. (Four replicates for each group); B–I) Verification of osteogenic gene expression of eight differential miRNAs via respective inhibitors and mimics; J) Verification of osteogenesis capacity of hBMSCs with the inhibitor and mimic of eight different miRNAs via alizarin red staining; K) The binding site prediction through targetscan of cbfb and miR‐222‐5p; L,M) The luciferase result of miR‐222‐5p and cbfb. The data are presented as mean ± SD, *n* = 4, and ^***^ *P* < 0.001.

To verify the miRNA sequencing results, RT‐PCR was performed in parallel (**Figure** **6**A). The results indicated that the expression levels of miR‐222‐5p, miR‐122‐5p, miR‐214‐5p, and miR‐30b‐5p were significantly different among the formerly identified eight miRNAs. Furthermore, we silenced and activated these differentially expressed miRNAs using miRNA mimics and inhibitors, respectively. In vitro tests, including ARS and qPCR, were performed to investigate the primary miRNAs mediating differences in the osteogenic potential of BMSCs. The results showed that miR‐222‐5p mimics significantly decreased the osteogenic capacity (ARS staining) via the expression of osteogenic genes, including Col1, Ocn, Alp, and Runx2 (Figure 6B–I). The knockdown of miR‐222‐5p abolished this effect. This finding indicates that the 90° microfiber‐mediated osteogenic differentiation of BMSCs is associated with miR‐222‐5p expression.

Furthermore, among the top 50 differentially expressed genes in the mRNA sequencing results (upregulation: fold change > 1.5, ^*^ *p* < 0.05; downregulation: fold change < 0.5, ^*^ *p* < 0.05), we identified Runx2 and cbfb as significantly related to osteogenesis, ECM formation, and focal adhesion (Figure 5E, red arrow). In our study, the microfibers mimicked the fibrous structure of natural ECM. Previously, Runx2 was shown to be closely related to the ECM, including cell‐ECM interactions, ECM production, and metabolism.^[^ ^18^ ^]^ As a co‐effector of Runx2, cbfb plays a critical role in the co‐activation of Runx2, which is required for skeletal development, effective DNA binding, and transcriptional activation of Runx2.^[^ ^19^ ^]^ These findings indicate that angled microfibers mimic the natural geometric ECM structure, regulate cellular behavior, and determine cell fate through miRNA‐mRNA interactions. As miRNAs regulate gene expression via pairing and degrading target mRNAs,^[^ ^20^ ^]^ we analyzed the corresponding mRNAs of these differentially expressed miRNAs, as shown in Figure 5I,J.

To investigate the downstream targets of miR‐222‐5p, we performed mRNA prediction via TargetscanHuman8.0 (https://www.targetscan.org/vert_80/). The results of mRNA prediction showed that cbfb was the direct downstream target of miR‐222‐5p (Figure 6K, site type = 8mer). This was verified using a luciferase assay (Figure 6L,M). Taken together, these results indicate that angled microfibers regulate miR‐222‐5p/cbfb to affect ECM interactions and metabolism and determine cell fate.

Geometric angles play important roles in cellular functions aspects and such angles are common in natural topological structures. However, the cellular‐level‐specific regulation of the formation of symbiotic ecological niches activated by mimicking angled structures at the cellular scale has not yet been systematically revealed. Collectively, these results indicate a potential angle‐mediated cbfb‐Runx2 pathway activation mechanism for osteogenesis. The cbfb/Runx2 axis has been widely reported to direct stem cell differentiation toward osteoblasts and cell‐ECM interactions.^[^ ^21^ ^]^ More importantly, cbfb activation is responsible for the osteogenesis of Runx2.^[^ ^22^ ^]^ Therefore, we hypothesized that angle‐mediated osteogenesis could regulate the expression of cbfb and Runx2, ultimately affecting ECM interactions and metabolism and modulating cell fate (e.g., osteogenesis).

As previously reported, the repair of tendon cell injury during osteogenic differentiation is blocked by miR‐222‐5p.^[^ ^23^ ^]^ By comparing the corresponding mRNA of miR‐222‐5p with the differentially expressed mRNA in our study, we identified cbfb as the primary target. It is a critical co‐effector in the stabilization and DNA binding of the Runx family proteins.^[^ ^24^ ^]^ Furthermore, cbfb plays a critical role in chondrocyte differentiation and skeletal development by stabilizing Runx2 and Runx3.^[^ ^25^ ^]^ A previous study reported that ECM stiffness repressed the Yap/Runx2 pathway to control VEGF secretion and neuroblastoma angiogenesis.^[^ ^26^ ^]^ Furthermore, another investigation revealed that PPARγ/Runx2 signaling pathway regulates the dynamic equilibrium of the chondrocyte ECM and degree of mineralization.^[^ ^27^ ^]^ Compared to other spatial topography angles (Figure 4), microfibers with a 90° topology significantly promoted the expression of osteogenic gene proteins in BMSCs. Taken together, microfibers with a 90° topology provide a geometric construction that fits the biological requirement to enhance the osteogenic differentiation of BMSCs via the miR‐222‐5p/cbfb/Runx2 axis.

### 2.6 In Vivo Bone Regeneration Evaluation

**Figure 7:**
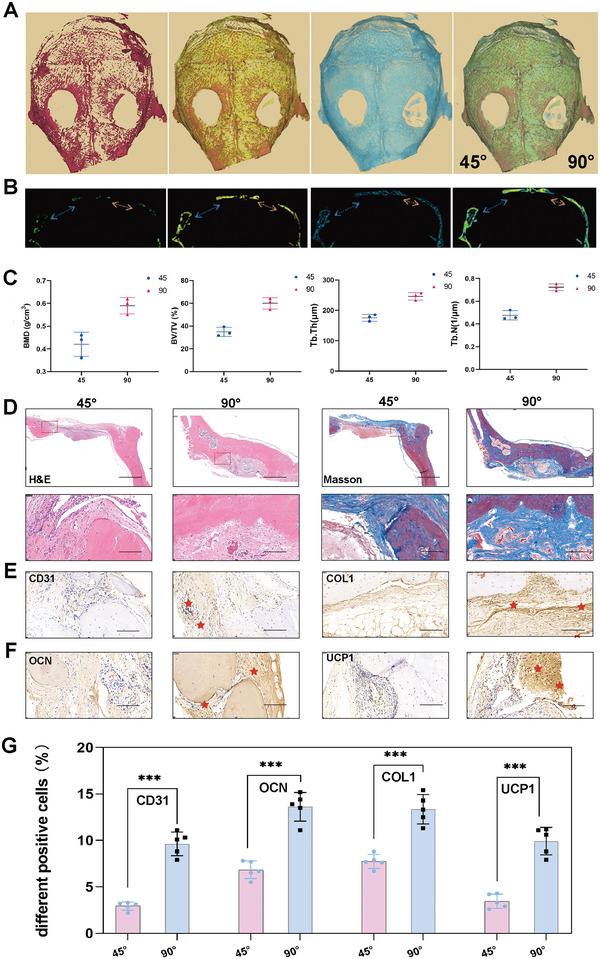
In vivo comparison of osteogenic performance of 45° and 90° microfibers. A) 3D reconstruction of cranial defects at 3 months after different scaffolds implantation (45° and 90°) (Red‐high density, yellow‐medium density, blue‐osteoid, and green‐newly formed bone); B) Micro‐CT images of cranial defect areas at 3 months post‐operation with different diameter (blue arrow‐45° and orange arrow‐90°), scale bar: 2 mm; C) Quantitative fundamental parameters in newborn osseous tissue based on the histomorphometric micro‐CT analysis (BMD: bone mineral density; BV/TV: bone volume/tissue volume; Tb.Th: trabecular thickness; Tb.N: trabecular number); D) HE and Masson staining of craniums after implanted with 45°/90° microfibers at 3 months and magnified images of the red rectangle, scale bar: 1 mm and 200 µm; E,F) The immunohistochemistry of CD31, OCN, COL1, and UCP1 in 45° and 90° microfibers, scale bar: 200 µm; G) Quantitative fundamental parameters of the positive cells of CD31, OCN, COL1, and UCP1 in 45° and 90° microfibers. The data are presented as mean ± SD, *n* = 5, and ^***^ *P* < 0.001.

**Figure 8:**
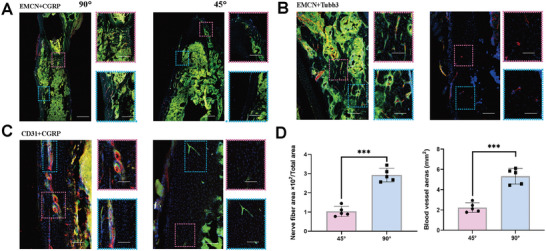
In vivo comparison of neurovascular performance of 45° and 90° microfibers. A–C) The immunofluorescence staining of EMCN, CGRP, CD31, and Tubb3 to identify nerve growth and angiogenesis with 45°/90° at 3 months, scale bar: 200 and 50 µm. D) Quantitative fundamental parameters of nerve finer and blood vessel areas. The data are presented as mean ± SD, *n* = 5, and ^***^ *P* < 0.001.

To evaluate the osteogenic potential of microfibers in vivo, the large cranial defect model was constructed in male C57 BL/6 mice based on previous studies.^[^ ^28^ ^]^ 3D reconstruction of micro‐CT scans and further analysis were performed at 3 months after surgery, including the 45° and 90° microfibers implantation. Cranial defects and residual scaffolds were more obvious in the 45° group than in the 90° group, indicating better bone regeneration by the 90° microfibers (**Figure** **7**A), which is consistent with the 3D‐printed bioglass in our previous studies.^[^ ^29^ ^]^ Moreover, the micro‐CT image of harvested craniums showed markedly increased calcified tissue surrounding the 90° microfibers with a shorter gap in the bone defect, which confirmed the intimate relationship between bone regeneration and the angle of the microfibers (Figure 7B). The parameters in Figure 7C show better bone regeneration and neovasculogenic performance for the 90° microfibers. Hematoxylin & eosin (H&E) and Masson's trichrome staining (Figure 7D) were conducted to evaluate the histological results of the 90° microfibers in vivo. The newly mineralized bone and the unmineralized bone were tightly combined with the residual fibers implanted with the 90° microfibers in the defect area. In contrast, the residual microfibers were evident in the 45° group with less new osteoid tissue. The 90° microfibers exhibited improved biodegradation and bone regeneration. In addition, immunohistochemical analysis of CD31, OCN, COL1, and UCP1 (Figure 7E–G) revealed new bone formation with different microfibers, confirming the better bone regeneration ability of the 90° microfibers. Immunofluorescence staining for EMCN, CGRP, CD31, and Tubb3 was performed to identify nerve growth and angiogenesis. The densities of nerve fibers and blood vessels in the 90° microfibers were higher than those in the 45° microfibers, suggesting superior neural development and angiogenesis (**Figure** **8**). We noticed that the in vitro data showed no significant difference in the growth of SCs among the different fiber angles. The data in vivo may be attributed to the complexity of bone tissue, where more activated osteoblast cells or BMSCs secrete cytokines, including NGF and VEGFa, to support nerve fiber ingrowth.^[^ ^30^ ^]^

To the best of our knowledge, no study has investigated the role of angle‐related geometric construction in bone tissue regeneration or the synergistic effects of bone and neural development. Electrospinning can boost vascularized bone regeneration based on self‐features and drug loading.^[^ ^31^ ^]^ Various multifunctional materials exhibit different capabilities and their properties play decisive roles in bone regeneration.^[^ ^32^ ^]^ Therefore, we constructed 3D‐printed microfibers with different geometric angles using the NFEP technique to investigate the regulatory mechanisms of the geometric angles on stem cell function and bone regeneration. With the change in the topography angle, the efficacy of bone regeneration was substantially boosted, with significantly increased bone mineral density (BMD), trabecular bone volume fraction (BV/TV), trabecular thickness (Tb.Th), and trabecular thickness (Tb.N). To provide further explore the effect of neural development on bone reconstruction, we conducted immunofluorescence staining for EMCN, CGRP, CD31, and Tubb3 to estimate nerve growth and angiogenesis using the 45° and 90° fibers. Collectively, these results indicate that angular change was the principal factor modulating bone regeneration in our study.

**Figure 9:**
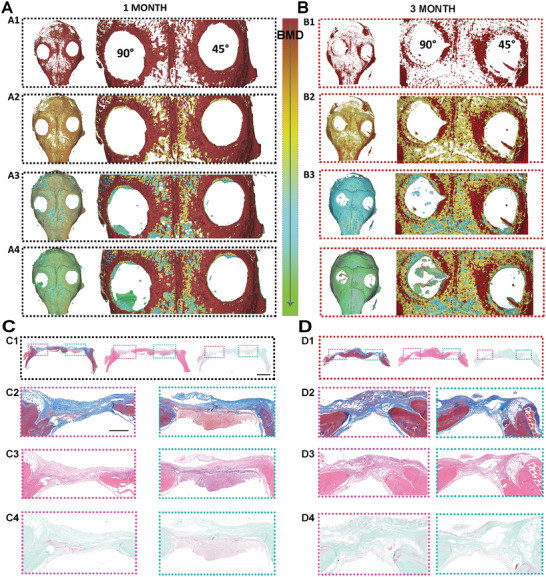
In vivo comparison of different time periods osteogenic performance of 45° and 90°. A) 3D reconstruction of different density bone at 1 month after different scaffolds implantation (45° and 90°) (A1‐high density, A2‐medium density, A3‐osteoid, and A4‐newly formed bone); B) 3D reconstruction of different density bone at 3 months after different scaffolds implantation (45° and 90°) (B1‐high density, B2‐medium density, B3‐osteoid, and B4‐newly formed bone). C) Histological staining of craniums with Masson, H&E, and Goldner implanted with 45°/90° at 1 month. Scale bar is 2 mm (C2‐Magnified images of Masson, C3‐Magnified images of H&E, and C4‐ Magnified images of Goldner. Scale bar is 1 mm) D) Histological staining of craniums with Masson, H&E, and Goldner implanted with 45°/90° at 3 months (D2‐Magnified images of Masson, D3‐ Magnified images of H&E, and D4‐ Magnified images of Goldner).

A comparison of osteogenic performance at different time points (1 and 3 months) with the 45° and 90° microfibers was also conducted (**Figure** **9**A,B). The bone mineral density gradually decreased, with color variability ranging from red to green. Material‐guided bone regeneration with the newly formed osteoid and collagen manifested the desired therapeutic effect with the 90° microfibers, confirming the osteoconductivity, osteoinductivity, and neovascularization capacity of the new platform. The 90° microfibers promoted new bone regeneration during the early stages and final bone reconstruction. H&E, Masson's trichrome, and Goldner staining (Figure 9C,D) were conducted to evaluate the histological effects of the 90° microfibers in vivo. The newly mineralized bone and the unmineralized bone were tightly combined with the residual scaffolds implanted with 90° microfibers, indicating excellent bone regeneration ability. Angled fibers mimic the natural structure of the ECM and provide a favorable microenvironment for bone regeneration, angiogenesis, and neural development.

Angles and structures are inherently present in natural bone tissues. For example, the anteversion angle of the femoral neck is 15° and the neck‐shaft angle of the femoral neck is 130°. These angles have inherent significance, and an abnormal angle causes limb deformity and dysfunction. Microscopically, the shape of type H vessels at the metaphysis is tortuous, providing more nutrition and oxygen to the epiphyseal plate.^[^ ^33^ ^]^ The Haversian canals, which contain blood vessels and nerves, are mainly parallel to the axis of the long shaft, whereas thinner Volkmann canals are usually more perpendicular to the long axis of the long shaft, forming a complex vascular network of bone tissue.^[^ ^34^ ^]^

Cells, as elementary units of bone tissue, continuously sense the mechanical cues of the microenvironment and make self‐adjustments.^[^ ^35^ ^]^ Specific tomography and angled structures play important roles at the cellular level. Osteogenic differentiation is triggered by different biophysical and chemical cues. During biophysical cue‐stimulated osteogenic differentiation, changes in focal adhesion behavior and cytoskeletal reorganization occur first. These alterations subsequently activate specific miRNA‐mRNA crosstalk. In our study, we employed miRNA and mRNA sequencing to reveal the role of the miR‐222‐5p/cbfb/Runx2 axis in the angle‐induced activation of BMSC osteogenesis via scaffolds. The 90° microfiber structure deforms the BMSCs to a more expanded morphology through cell skeleton reorganization, directing the cell fate toward osteogenesis. Finally, a rat cranial defect model demonstrated the specific performance of the scaffolds in terms of new bone formation and nerve ingrowth. Our study provides guidelines for designing simple, efficient, and personalized bone grafts with simultaneous osteogenesis.

## 3 Conclusion

In summary, this study utilized an artificial, fabricated, angled structure to provide a suitable microenvironment topography to induce osteogenic differentiation of BMSCs, and promote bone regeneration. We found that the miR‐222‐5p/cbfb/Runx axis mediates osteogenic differentiation of BMSCs induced by geometric features. Overall, this study systematically elucidates how geometric architecture can promote stem cell differentiation and function for structural bone regeneration.

## 4 Experimental Section

### Materials

PCL (Mw = 80 000) was obtained from Jinan Daigang Co., Ltd. (China). 1,1,1,3,3,3‐Hexaflfluoro‐2‐propanol (HFIP) was obtained by Aladdin Industrial Co., Ltd. (Shanghai, China). Cells were supplied by the Shanghai Institute of Biochemistry and Cell Biology (Shanghai, China). The cell culture reagents were acquired from Gibco (Waltham, MA, USA). Unless otherwise mentioned, all other chemicals and reagents were obtained from Sigma–Aldrich (St. Louis, MO, USA).

### Fabrication and Characterization of 3D Microfiber Patterns

In this study, NFEP was developed to configure multi‐scale microfiber patterns, as previously reported.^[^ ^12^ ^]^ All the designed microfiber patterns were programmed using *g*‐code. Briefly, the PCL solution (10% in HFIP) was loaded into a syringe, and the mobile stage with *X‐Y‐Z* linear motion was used to collect microfibers by modulating interfiber overlay angles (30°, 45°, 60°, 75°, and 90°) during NFEP. The optimal near‐field electrostatic printing conditions for the 3D microfiber patterns were determined: the applied voltage: 1.5 kV, the flow rate: 0.5 µL min^−1^, and the collection distance: 2 mm.

The macroscopic appearance and topological morphology of these microfibers were characterized using a digital camera (Canon, Tokyo, Japan) and SEM (FEI, Hillsboro, OR, USA), respectively. The average microfiber diameter, gap length, and roughness were measured using ImageJ and Gwyddion analyses.

### Culture of HUVECs and SCs on Microfiber Patterns

To improve the cell adhesion of microfibers, the various microfiber patterns were treated with Polylysine (PLL) for 20 min at 37 °C in a humidified at incubator. After washing with PBS to remove free PLL, the PLL‐encapsulated PCL microfibers were obtained. HUVECs and SCs, which widely exist in tissues, were chosen to further demonstrate the influence of fiber structures of different diameters on cellular biological functions. HUVECs or SCs with a density of 1.0 × 10^4^ cells per well were co‐cultured with the different microfiber patterns in a humidified incubator under 5% CO_2_ at 37 °C. The cells were cultured in a high‐sugar medium containing 10% FBS and 1% penicillin/streptomycin, and the medium was refreshed every 2 d.

After 1, 3, and 5 d of culture, cytotoxicity was evaluated using the CCK‐8 assay (K1018; Apexbio, Houston, TX, USA). After 5 d of culture, the cells were fixed with 4% paraformaldehyde (PFA) for 20 min, permeabilized with 0.1% Triton X‐100 for 5 min, and blocked with 2% BSA for 30 min at room temperature. HUVECs and SCs were treated with antibodies against CD31 (1:100, Abcam) and NGF (1:100, Abcam) overnight at 4 °C, respectively, followed by treatment with the Alexa Fluor 488 goat anti‐rabbit IgG H&L (1:200, Abcam) for 1 h at room temperature. Alexa Fluor 594 phalloidin and DAPI were used to label actin and nuclei, respectively. Samples were observed under a Zeiss laser scanning confocal microscope (LSCM; LSM800, Zeiss, Oberkochen, Germany). The average fluorescence intensities of positive CD31 and NGF staining were determined using Image J.

### Culture of BMSCs on Microfiber Patterns

The rat BMSCs were extracted from 4‐week‐old male SD rats (Shanghai Jihui Experimental Animal Center, Shanghai, China) according to the previously described protocol. Human BMSCs were obtained from the cell bank of the Chinese Academy of Sciences (Shanghai, China). To investigate whether different microfibers could affect the differentiation of BMSCs by modulating the interfiber overlay angles, BMSCs with a density of 3.0 × 10^4^ cells per well were co‐cultured with different microfiber patterns. After 14 d, immunofluorescence staining (OPN) was performed as previously described. Briefly, after the cells were fixed, permeabilized, and blocked, they were incubated with a primary antibody against OPN (ab63856; Abcam), followed by incubation with secondary antibodies, phalloidin, and DAPI successively. Finally, samples were observed via LSCM. Additionally, Alizarin Red Staining was used to evaluate ECM mineralization.

### RT‐qPCR and Western Blotting

For gene expression analysis, total RNA was extracted from various cells (HUVECs, SCs, and human BMSCs) on various microfiber patterns using an RNA extraction kit (Gibco), as previously described.^[^ ^36^ ^]^ Following the manufacturer's instructions, the extracted RNA was reverse‐transcribed into cDNA via the PrimeScript RT reagent kit (Takara, Kusatsu, Japan). The qRT‐PCR was carried out using a SYBR Green qRT‐PCR kit (Takara). The corresponding primer sequences were showed in Table S1 (Supporting Information). Western blotting was performed to detect the expression of the proteins involved in rat BMSC osteogenesis. Briefly, the total cellular protein was extracted using a BCA protein kit (Servicebio, Wuhan, China). After treatment with 10% sodium dodecyl sulfate‐polyacrylamide gel electrophoresis (Servicebio), western blots were transferred to a PVDF membrane (Millipore). The membrane was blocked with 5% BSA and then incubated with antibodies against Col1a (Abcam, 1:1000), OPN (Abcam, 1:1000), OCN (Abcam, 1:1000), VEGF (Abcam, 1:1000), and Actin (Abcam, 1:1000). After treatment with the corresponding secondary antibody (1:3000, Abcam) for 30 min, the blots were detected and determined by the chemiluminescence system and ImageJ software.

### High‐Throughput Sequencing

The mRNA expression level in human BMSCs was quantified by high‐throughput sequencing after 24 h treatment with 45° and 90° microfibers. Following the manufacturer's instructions, total RNA was isolated using an RNAqueous‐Midi Kit (AM1911, Thermo Fisher Scientific). To analyze differentially expressed miRNAs (DEMs), high‐throughput sequencing was carried out in triplicate using an Illumina HiSeq X Ten Second Generation Sequencing Platform (Seq‐Health Co., Wuhan, China). The DEM with significant differences between the two groups were identified by fold‐change filtering and volcano plot. A two‐fold difference in the expression threshold, with a *P*‐value < 0.01, was selected as the cut‐off for the identification of DEMs.

### Luciferase

BMSCs were routinely cultured at 37 °C with 5% CO_2_, they were inoculated at 1.5 × 10^4^ cells per well in a 96‐well plate with a total volume of 100 µL per well, and incubated at 37 °C for 24 h. The 10 µL OPTI‐MEM medium was used to dilute miRNA mimics or mimic NC, 15 µL OPTI‐MEM medium to dilute the target gene 3′UTR double reporter gene or mutation vectors, and 25 µL OPTI‐MEM medium to dilute 0.25 µL Lipofectamine 2000 reagent for 5 min, then the three were mixed for a total of 50 µL. Before adding the plasmid and mimics to the cells, 50 µL of the medium was aspirated per well, and 50 µL of the mixture was added to a total volume of 100 µL per well. The mimic transfection concentration was 50 nm, the plasmid concentration was 250 ng per well, and three replicate wells were used for each group. One hundred microliters of fresh medium were added after 6 h. Before fluorescence detection, the kit's luciferase substrate was mixed with luciferase buffer solution, stored at −80 °C, and balanced to room temperature before use. The stop & Glo buffer solution was stored at −80 °C, returned to room temperature to use, and an appropriate amount was added. After 48 h transfection, 1 X PBS (35 µL per well) was added into the culture medium, then luciferase substrate (35 µL per well) was added, and the fluorescence value was measured with shaking for 10 min. Thirty microliters of stop reagent was added, the mixture was oscillated for 10 min, and the fluorescence value was measured using a fluorometer.

### Surgical Procedures of Cranial Bone Defect and Scaffold Implantation

All animal procedures were agreed by the Animal Ethics Committee of Shanghai Jiao Tong University Affiliated Sixth People's Hospital (Ethical Clearance number: 2021‐0334). Twenty‐four male C57 BL/6 mice (25–30 g) were used in the experiments and randomly divided into four groups (*n* = 6): i) 45°+90° 1m group; ii) 45°+90° 3m group; iii) 90°+45° 3m group, and iv) control group. The animals were anesthetized by an intraperitoneal injection of pentobarbital (Nembutal). Two 2 mm calvarial defects were created using an electric trephine (Nouvag AG, Goldach, Switzerland) to ensure that the dura mater remained uninjured. The defects were filled with 45° and 90° microfibers, respectively (Φ 2 × 2 × 0.2 mm). Postoperatively, each animal received an intramuscular injection of antibiotics. None of the mice exhibited adverse reactions to the implants during the experiments. The mice were successively euthanized by an overdose of the anesthetic after 1 and 3 months. The craniums of mice in group (i) were harvested at 1 month, and those of mice in groups (ii)–(iv) were harvested at 3 months.

### Micro‐CT Analysis

The harvested samples were scanned using a micro‐CT scanner (Skyscan, Kontich, Belgium). The scanning energy was 35 kV/100 µA and the voxel size was 18 µm. Mimics software was used to obtain reconstructed images. Bone mineral density (BMD), trabecular thickness (Tb.Th), trabecular bone volume fraction (BV/TV), and trabecular thickness (Tb.N) were measured by the auxiliary software of the mCT‐80 system.

### Histological Analysis of Newborn Bone Tissue

The harvested samples were obtained after 1 month and 3 months and were sequentially fixed with 4% paraformaldehyde for 72 h, decalcified with EDTA for 1 month, and embedded with paraffin. Finally, H&E, Masson, and Goldner trichrome staining were performed to evaluate the newborn osseous tissue. In addition, immunohistochemical staining of CD31, COL1, OCN, and UCP1 (Abcam) were also performed to assess the subchondral trabecular structures. Microscope (LEICA DM 4000) and Image J software were used for image capture and quantitative analysis, respectively.

### Statistical Analysis

All data were presented as mean ± standard deviation. Data analysis was analyzed using Origin 8.5 software, and statistical differences between data were analyzed using One‐way analysis of variance (ANOVA) or and *t*‐test. ^*^ *P* < 0.05, ^**^ *P* < 0.01, and ^***^ *P* < 0.001 were considered statistically significant, moderately significant, and highly significant, respectively.

## Conflict of Interest

The authors declare no conflict of interest.

## Supporting information

Supporting Information

## Data Availability

The data that support the findings of this study are available from the corresponding author upon reasonable request.
