# Supplementary material for: Geometric Angles and Gene Expression in Cells for Structural Bone Regeneration
Source: Adv Sci (Weinh). 2023 Sep 29;10(32):2304111. doi: 10.1002/advs.202304111 (PMC10646237; doi:10.1002/advs.202304111)
Supplement: Supplementary file 1 — Supporting Information [file ADVS-10-2304111-s001.pdf]

ADVANCED SCIENCE
Open Access

Supporting Information

for *Adv. Sci.*, DOI 10.1002/advs.202304111

Geometric Angles and Gene Expression in Cells for Structural Bone Regeneration

Juan Wang, Qianhao Yang, Qimanguli Saiding, Liang Chen, Mingyue Liu, Zhen Wang, Lei Xiang, Lianfu Deng, Yixuan Chen and Wenguo Cui**

Geometric Angles and Gene Expression in Cells for Structural Bone Regeneration

Juan Wang, Qianhao Yang, Qimanguli Saiding, Liang Chen, Mingyue Liu, Zhen Wang, Lei Xiang, Lianfu Deng, Yixuan Chen, Wenguo Cui**

Dr. J. Wang, Dr. Q. Saiding, Dr. L. Chen, Dr. M. Liu, Z. Wang, L. Xiang, Prof. W. Cui

Department of Orthopaedics, Shanghai Key Laboratory for Prevention and Treatment of Bone and Joint Diseases, Shanghai Institute of Traumatology and Orthopaedics, Ruijin Hospital, Shanghai Jiao Tong University School of Medicine, 197 Ruijin 2nd Road, Shanghai 200025, P. R. China.

E-mail: wgcui80@hotmail.com; wgcui@sjtu.edu.cn (W. Cui)

Dr. Q. Yang, Dr. Y. Chen

Department of Orthopedic Surgery, Shanghai Jiao Tong University Affiliated Sixth People's Hospital, Shanghai 200233, P. R. China

F-mail: cyxwlp0987@alumni.sjtu.edu.cn (Y. Chen)

Keywords: Geometric angles; Microfibers; Stem cell function; microRNA sequencing; bone regeneration;

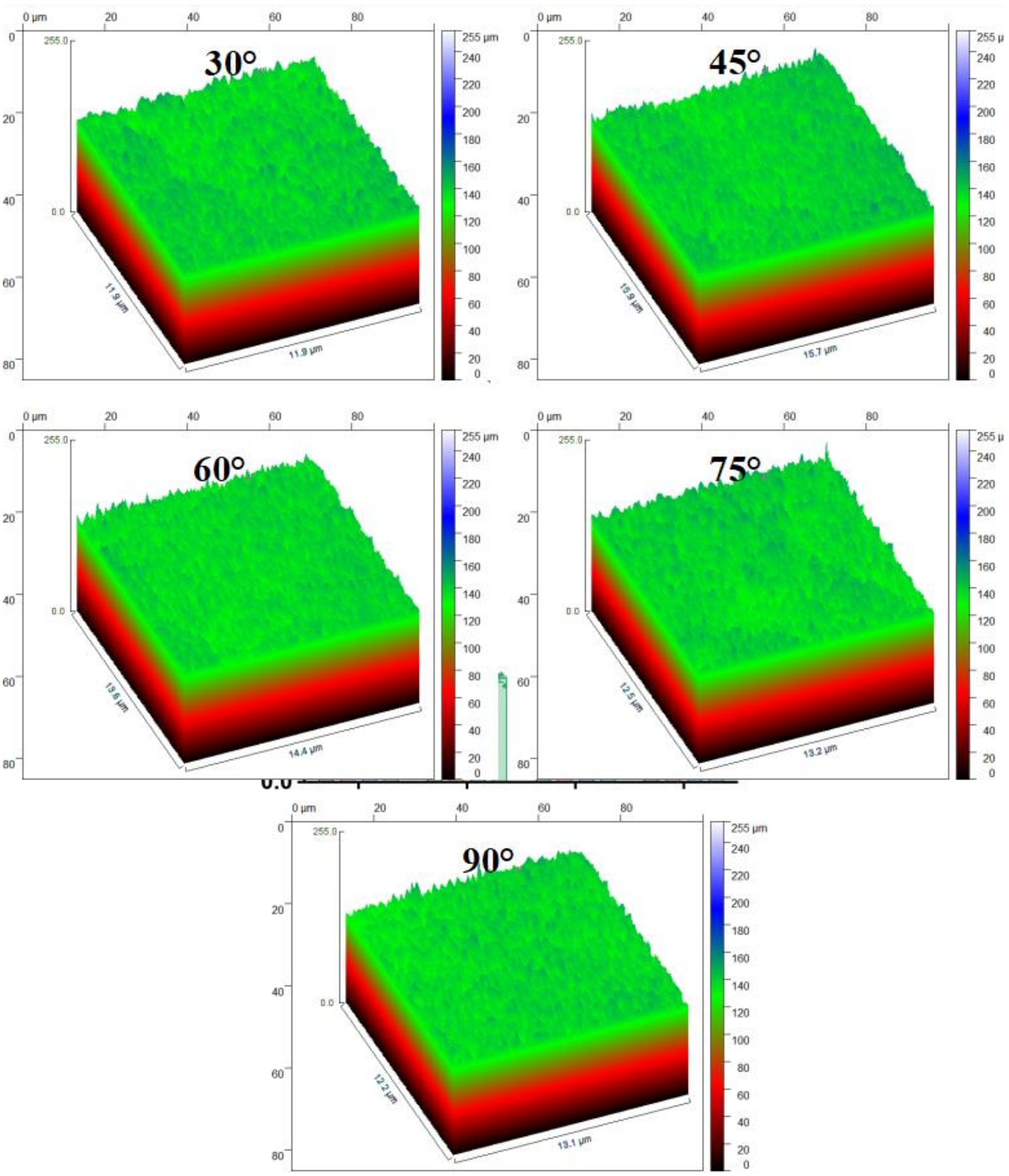

Figure. S1 Surface roughness of the microfiber patterns.

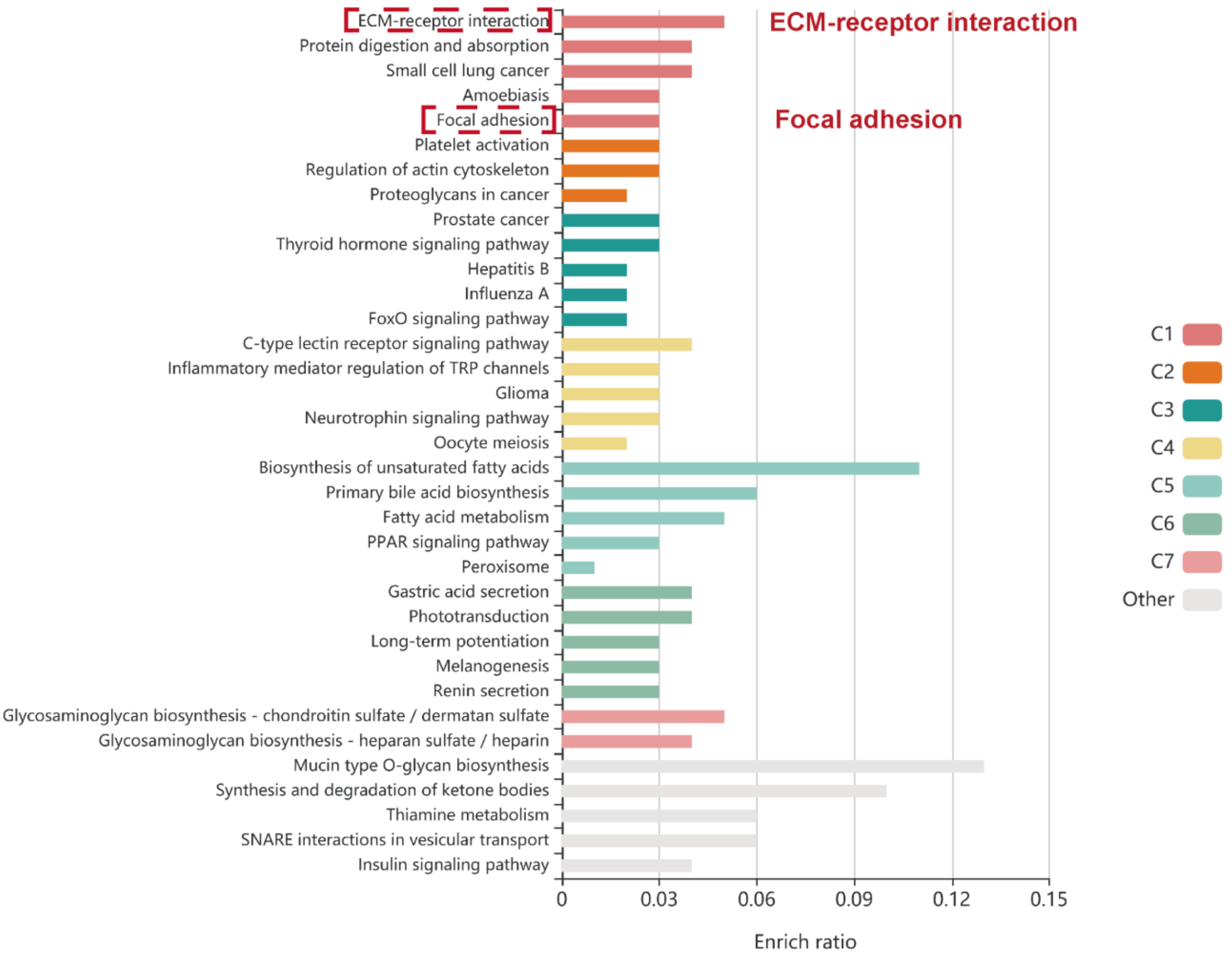

Figure. S2 KEGG enrichment analysis of miRNA sequencing result indicated ECM-receptor interaction and focal adhesion are among the most related biological process.

Table S1. Primer sequences of the genes for qRT-PCR analysis

Primers	Forward sequence (5'-3')	Reverse sequence (5'-3')
**VEGF**	GGAGGGCAGAATCATCACGA	GCTCATCTCTCCTATGTGCTGG
**CD31**	AGCTAACAGTCATTACGGTCAC	TCTGCTTTCCACGGCATCAGG
**PMP22**	CTTCGTCTCCACCATCGTCA	GACCAGCAAGGATTTGGAAGAC
**NGF**	ACTTCAGCATTCCCTTGACACA	AAATCCAGAGTGTCCGAAGAGG
**OPN**	TGAAACGAGTCAGCTGGATG	TGAAATTCATGGCTGTGGAA
**OCN**	AGCCTTTGTGTCCAAGCA	CCAGCCATTGATACAGGTAG
**ALP**	GGAGGTCGGATAGTTTCGAT	GCTGGTTGTCGTTCGCTTGA
**Runx2**	TAATCTCCGCAGGTCACTAC	CTGAAGAGGCTGTTTGATG
**CoI I**	GACATCCCACCAATCACCTG	CGTCATCGCACAACACCTT
